# A 3D Sensor Based on a Profilometrical Approach

**DOI:** 10.3390/s91210326

**Published:** 2009-12-21

**Authors:** Jesús Carlos Pedraza-Ortega, Efren Gorrostieta-Hurtado, Manuel Delgado-Rosas, Sandra L. Canchola-Magdaleno, Juan Manuel Ramos-Arreguin, Marco A. Aceves Fernandez, Artemio Sotomayor-Olmedo

**Affiliations:** Facultad de Informatica, Universidad Autonoma de Queretaro, Av. de las Ciencias S/N, Juriquilla, C.P. 76230, Queretaro, Mexico; E-Mails: efren.gorrostieta@uaq.mx (E.G.-H.); mdelgado80@hotmail.com (M.D.-R.); sandracanchola@yahoo.com (S.L.C.-M.); jramos@uaq.mx (J.M.R.-A.); marco.aceves@uaq.mx (M.A.A.F.); artemio.sotomaror@uaq.mx (A.S.-O.)

**Keywords:** 3D reconstruction, image processing, segmentation, Fourier transform, wavelet transform

## Abstract

An improved method which considers the use of Fourier and wavelet transform based analysis to infer and extract 3D information from an object by fringe projection on it is presented. This method requires a single image which contains a sinusoidal white light fringe pattern projected on it, and this pattern has a known spatial frequency and its information is used to avoid any discontinuities in the fringes with high frequency. Several computer simulations and experiments have been carried out to verify the analysis. The comparison between numerical simulations and experiments has proved the validity of this proposed method.

## 1. Introduction

In the last three decades, the idea of extracting the 3D information of a scene from its 2D images has been widely investigated. Several contact and non-contact measurement techniques have been employed in many science and engineering applications to compute the 3-D surface of an object. Basically, the aim is to extract the useful depth information from an image in an efficient and automatic way. Then, the obtained information can be used to guide various processes such as robotic manipulation, automatic inspection, reverse engineering, 3D depth map for navigation and virtual reality applications [1]. Among all the diverse methodologies, one of the most widely used is the fringe projection. Fringe processing methods are widely used in non-destructive testing, optical metrology and 3D reconstruction systems. Some of the desired characteristics in these methods are high accuracy, noise-immunity and fast processing speed.

Scarcely used fringe processing methods are the well-known Fourier Transform Profilometry (FTP) method [2] and Phase-shifting Interferometry [3]. It is also well-established that one of the main challenges for these methods is the wrapped phase information problem. The first algorithm for this purpose was proposed by Takeda and Mutoh in 1982 [2]. Later Berryman [4] and Pedraza [5-7] proposed a modified Fourier Transform Profilometry by carrying out global and local analyses in the phase unwrapping. Then, unwrapping algorithms (temporal and spatial) were introduced and modified [8-10]. Another solution is to extract the information by the use of a wavelet transform.

Due to the fact that the wavelet transform offers multiresolution in time and space frequency, it is a tool that offers advantages over the Fourier transform [11,12]. The computation in the method can be carried out by analyzing the projected fringe patterns using a wavelet transform. Mainly, this analysis consists of demodulating the deformed fringe patterns and extracting the phase information encoded into it and hence the height profile of the object can be calculated, quite similar to Fourier transform.

Different wavelet algorithms are used in the demodulation process to extract the phase of the deformed fringe patterns. They can be classified into two categories: phase estimation and frequency estimation techniques.

The phase estimation algorithm employs complex mother wavelets to estimate the phase of a fringe pattern. The extracted phase suffers from 2π discontinuities and a phase unwrapping algorithm is required to remove these 2π jumps. Zhong *et al.* [11] have applied Gabor wavelets to extract the phase distribution where a phase unwrapping algorithm is required.

The frequency estimation technique estimates the instantaneous frequencies in a fringe pattern, which are then integrated to estimate the phase. The phase extracted using this technique is continuous; consequently, phase unwrapping algorithms are not required. Complex or real mother wavelets can be used to estimate the instantaneous frequencies in the fringe pattern. Dursun *et al.* [13] and Afifi *et al.* [14] have used Morlet and Paul wavelets, respectively, to obtain the phase distribution of projected fringes without using any unwrapping algorithms. Also, Gdeisat *et al.* [15] have proposed a 1D continuous wavelet transform approach to retrieve phase information in temporally and spatially fringe patterns, moreover, several algorithms were used for ridge extraction in the phase of the fringe patters.

Most of the previous research is focused on using the Fourier and wavelet transforms separately to obtain the 3D information from an object; pre-filtering the images, extracting the phase information of fringe patterns, using phase unwrapping algorithms, and so on.

In the present research, a simple profilometrical approach to obtain the 3D information from an object is presented. Here, the spatial frequency of the projected fringe pattern is obtained. The mathematical description to obtain the spatial frequency is a contribution in this research. Then, a modified Fourier transform method of an extended 1D wavelet based profilometry is applied. Later, a robust phase unwrapping algorithm is developed and used to obtain the desired 3D information. The main contribution of this work is the methodology. In addition, this novel approach is compared with other similar research and the results are presented. In order to validate the methodology, some virtual objects were created for use in computer simulations and experiments.

## 2. Theoretical Background

As described in the previous section, there are several fringe projection techniques which are used to extract the three-dimensional information from the objects. In this section, a Modified Fourier Transform is explained and the Wavelet Profilometry is introduced.

### 2.1. Fourier Transform Profilometry

The image of a projected fringe pattern and an object with projected fringes can be represented by:

(1) $$g(x,y)=a(x,y)+b(x,y)\times \cos[2\times \pi f_{0}x+\phi (x,y)]$$

(2) $$g_{0}(x,y)=a(x,y)+b(x,y)\times \cos[2\times \pi f_{0}x+\phi_{0}(x,y)]$$

where *g*(*x,y*) and *g*_0_(*x,y*) are the intensities of the images at the point (*x,y), a*(*x,y*) represents the background illumination, *b*(*x,y*) is the contrast between the light and dark fringes, *f_0_* is the spatial-carrier frequency and *φ*(*x,y*) and *φ_0_*(*x,y*) are the corresponding phase to the fringe and distorted fringe pattern, observed by the camera.

The phase *φ*(*x,y*) contains the desired information, whilst *a*(*x,y*) and *b*(*x,y*) are unwanted irradiance variations. The angle *φ*(*x,y*) is the phase shift caused by the object surface end the angle of projection, and its expressed as:

(3) $$\varphi (x,y)=\varphi_{0}(x,y)+\varphi_{z}(x,y)$$

where *φ_0_*(*x,y*) is the phase caused by the angle of projection corresponding to the reference plane, and *φ_z_*(*x,y*) is the phase caused by the object's height distribution.

**Figure 1.**
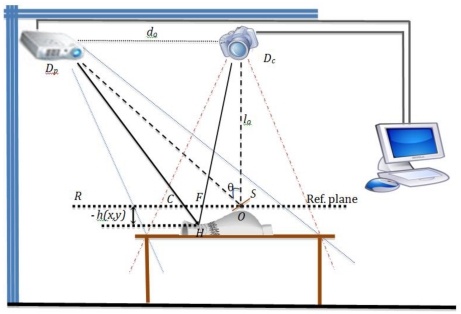
Experimental setup.

Considering Figure 1, we have a fringe which is projected from the projector, the fringe reaches the object at point H and will cross the reference plane at the point C. By observation, the triangles DpHDc and CHF are similar and since:

(4) $$\frac{CD}{-h}=\frac{d_{0}}{l_{0}}$$

This leads to the next equation:

(5) $$\varphi_{z}(x,y)=\frac{h(x,y)2\pi f_{0}d_{0}}{h(x,y)-l_{0}}$$

where the value of *h*(*x,y*) is measured and considered as positive to the left side of the reference plane. Equation 5 can be rearranged to express the height distribution as a function of the phase distribution:

(6) $$h(x,y)=\frac{l_{0}\phi_{z}(x,y)}{\phi_{z}(x,y)-2\pi f_{0}d_{0}}$$

#### 2.1.1. Fringe Analysis

The fringe projection Equation 1 can be rewritten as:

(7) $$g(x,y)=\sum_{n=-\infty }^{\infty }A_{n}r(x,y)\exp{\left(in\varphi (x,y)\right)}^{*}\exp(i2\pi nf_{0}x)$$

where *r*(*x,y*) is the reflectivity distribution on the diffuse object [3,4]. Then, a FFT (Fast Fourier Transform) is applied to the signal in the x direction only. Thus, the following equation is obtained:

(8) $$G(f,y)=\sum_{-\infty }^{\infty }Q_{n}(f-nf_{0},y)$$

where *Q_n_* is the 1D Fourier Transform of *A_n_ exp*[*inφ*(*x,y*)].

Here *φ*(*x,y*) and *r*(*x,y*) vary very slowly in comparison with the fringe spacing, then the Q peaks in the spectrum are separated from each other. It is also necessary to consider that if a high spatial fringe pattern is chosen, the FFT will have a wider spacing among the frequencies. The next step is to remove all signals with exception of the positive fundamental peak *f_0_*. The obtained filtered image is then shifted by *f_0_* and centred. Later, the IFFT (Inverse Fast Fourier Transform) is applied in the *x* direction only, as well as the FFT. Separating the phase part of the result from the rest we obtain:

(9) φz(x,y)=φ(x,y)+φ0(x,y)=Im{log(g^(x,y)g^0∗(x,y))}

It is observed that the phase map can be obtained by applying the same process for each horizontal line. The values of the phase map are wrapped at some specific values. Those phase values range between π and −π.

To recover the true phase it is necessary to restore the measured wrapped phase by an unknown multiple of 2π*f_0_* [16]. The phase unwrapping process is not a trivial problem due to the presence of phase singularities (points in 2D, and lines in 3D) generated by local or global undersampling. The correct 2D branch cut lines and 3D branch cut surfaces should be placed where the gradient of the original phase distribution exceeded π rad value. However, this important information is lost due to undersampling and cannot be recovered from the sampled wrapped phase distribution alone. Also, is important to notice that finding a proper surface, or obtaining a minimal area or using a gradient on a wrapped phase will not work and one could not find the correct branch in cut surfaces. From here, it can be observed that some additional information must be added in the branch cut placement algorithm.

#### 2.1.2. Phase Unwrapping

As mentioned earlier, the unwrapping step consists of finding discontinuities of a magnitude close to 2π, and then, depending on the phase change, 2π can be added or subtracted to the shape according to the sign of the phase change. There are various methods for doing the phase unwrapping, and the important factor to consider in this step is the abrupt phase changes in the neighbouring pixels. There are a number of 2π phase jumps between two successive wrapped phase values, and this number must be determined. This number depends on the spatial frequency of the fringe pattern projected at the beginning of the process.

This step is the modified part in the Fourier Transform Profilometry originally proposed by Takeda [3], which is considered as another contribution of this work. Another additional consideration is to carry out a smoothing before doing the phase unwrapping. This procedure will help to reduce the error produced by the unwanted jump variations in the wrapped phase map. Similar methods are described in [5]. Moreover, a modified Fourier Transform Profilometry method was used in [10] that included further analysis which considers local and global properties of the wrapped phase image.

### 2.2. Wavelet Transform

The wavelet transform (WT) is considered an appropriate tool to analyze non-stationary signals. This technique has been developed as an alternative approach to the most common transforms, such as Fourier transform, to analyze fringe patterns. Furthermore, WT has a multi-resolution property in both time and frequency domains which solves a commonly know problem in other transforms like the resolution.

**Figure 2.**
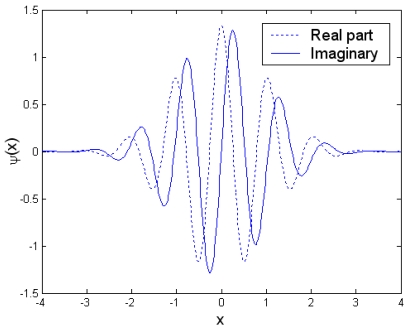
Complex Morlet wavelet.

A wavelet is a small wave of limited duration (this can be real or complex). For this, two conditions must be satisfied: firstly, it must have a finite energy. Secondly, the wavelet must have an average value of zero (admissibility condition). It is worth noting that many different types of mother wavelets are available for phase evaluation applications. The most suitable mother wavelet is probably the complex Morlet one [2]. The Morlet wavelet is a plane wave modulated by a Gaussian function, and is defined as:

(10) $$\psi (x)=\pi^{1/4}\exp(\text{icx})\exp(-x^{2}/2)$$

where c is a fixed spatial frequency, and chosen to be about 5 or 6 to satisfy an admissibility condition [17]. Figure 2 shows the real part (dashed line) and the imaginary part (solid line) of the Morlet wavelet.

The one-dimensional continuous wavelet transform (1D-CWT) of a row *f*(*x*) of a fringe pattern is obtained by translation on the x axis by b (with y fixed) and dilation by s of the mother wavelet ψ(x) as given by:

(11) $$W(s,b)=\frac{1}{\sqrt{s}}\int_{-\infty }^{\infty }f(x)\psi^{*}\left(\frac{x-b}{s}\right)dx$$

where * denotes complex conjugation and *W(s,b)* is the calculated CWT coefficients which refers to the closeness of the signal to the wavelet at a particular scale.

#### Wavelet Phase Extraction Algorithms

In this contribution, phase estimation and frequency estimation methods are used to extract the phase distribution from two dimensional fringe patterns. In the phase estimation method, a complex Morlet wavelet will be applied to a row of the fringe pattern. The resultant wavelet transform is a two dimensional complex array, where the phase arrays can be calculated as follows:

(12) abs(s,b)=|W(s,b)|

(13) $$\varphi (s,b)=\tan^{-1}\left(\frac{ℑ\{W(s,b)\}}{ℜ\{W(s,b)\}}\right)$$

To compute the phase of the row, the maximum value of each column of the modulus array is determined and then its corresponding phase value is found from the phase array. By repeating this process on all rows of the fringe pattern, a wrapped phase map results and an unwrapping algorithm is then needed to unwrap it.

In the frequency estimation method, a complex Morlet wavelet is applied to a row of the fringe pattern. The resultant wavelet transform is a two dimensional complex array. The modulus array can be found using Equation (12) and hence the maximum value for each column and its corresponding scale value can be determined. Considering that we are interested in the 1D signal:

(14) $$f(x)=a(x)+b(x)\cos(2\pi f_{0}x+\varphi (x))$$

Due to the fact that:

(15) $$\cos(x)=\frac{e^{i\theta }+e^{-i\theta }}{2}$$

We can re-write the Equation 14 as:

(16) $$f(x)=a(x)+b(x)\cos(\varphi (x))=a(x)+b(x)\frac{e^{i\varphi (x)}+e^{-i\varphi (x)}}{2}=a(x)+b(x)\frac{e^{i\varphi (x)}}{2}+b(x)\frac{e^{-i\varphi (x)}}{2}$$

Then, we consider the analytic function *f* in an open interval A, where *z_0_ ∈ A* and assuming that *D(z_0_;r) ⊂ A* → *f* can be decompose into Taylor series as:

$$f(z)=\sum_{k=0}^{\infty }\frac{f^{\left(k\right)}(z_{0})}{k!}{\left(z-z_{0}\right)}^{k}$$

Therefore:

(17) $$\varphi (x)=\varphi (x)+\varphi^{\prime }(x)(b)(x-b)+\frac{\varphi^{\prime \prime }(x)}{2!}{\left(x-b\right)}^{2}+\frac{\varphi^{\prime \prime \prime }(x)}{3!}{\left(x-b\right)}^{3}+\ldots$$

If :

(18) $$\varphi (x)=\varphi (b)+\varphi^{\prime \prime }(b)\approx 0,\varphi^{\prime \prime \prime }(b)\approx 0,{\varphi^{\prime }}^{v}(b)\approx 0,\cdots ,\varphi^{k}(b)\approx 0$$

then, the function can be reduced as:

(19) $$\varphi (x)=\varphi (b)+\varphi^{\prime }(b)(x-b)$$

Moreover, the Morlet Wavelet is defined as $\psi (x)=e^{i\varpi_{0}x}e^{-\frac{x^{2}}{2}}$, this wavelet will be applied to the mother wavelet:

$$W(s,b)=\frac{1}{s}\int_{-\infty }^{\infty }f(x)\psi^{*}\left(\frac{x-b}{s}\right)dx$$

If *s* = *1*, then:

(20) $$\begin{array}{l} W(s,b)=\frac{1}{s}\int_{-\infty }^{\infty }f(x)\psi^{*}\left(\frac{x-b}{s}\right)dx=\int_{-\infty }^{\infty }\left[a(x)+b(x)\frac{e^{i\varphi (x)}}{2}+b(x)\frac{e^{-i\varphi (x)}}{2}\right]\psi^{*}\left(\frac{x-b}{s}\right)dx=\\ =\int_{-\infty }^{\infty }a(x)\psi^{*}\left(\frac{x-b}{s}\right)dx+\int_{-\infty }^{\infty }b(x)\frac{e^{i\varphi (x)}}{2}\psi^{*}\left(\frac{x-b}{s}\right)dx+\int_{-\infty }^{\infty }b(x)\frac{e^{-i\varphi (x)}}{2}\psi^{*}\left(\frac{x-b}{s}\right)dx=\\ =\int_{-\infty }^{\infty }a(x)\psi^{*}\left(\frac{x-b}{s}\right)dx+\int_{-\infty }^{\infty }b(x)\frac{e^{i[\varphi (b)+\varphi^{\prime }(b)(x-b)]}}{2}\psi^{*}\left(\frac{x-b}{s}\right)dx+\int_{-\infty }^{\infty }b(x)\frac{e^{-i[\varphi (b)+\varphi^{\prime }(b)(x-b)]}}{2}\psi^{*}\left(\frac{x-b}{s}\right)dx=\\ =a\int_{-\infty }^{\infty }\psi^{*}\left(\frac{x-b}{s}\right)dx+\frac{b}{2}\int_{-\infty }^{\infty }\frac{e^{i[\varphi (b)+\varphi^{\prime }(b)(x-b)]}}{2}\psi^{*}\left(\frac{x-b}{s}\right)dx+\frac{b}{2}\int_{-\infty }^{\infty }\frac{e^{-i[\varphi (b)+\varphi^{\prime }(b)(x-b)]}}{2}\psi^{*}\left(\frac{x-b}{s}\right)dx \end{array}$$

By solving Equation 20, the following equation is obtained:

(21) $$a\sqrt{2\pi }e^{-\frac{1}{2}\varpi_{0}^{2}}+\frac{b}{2}\sqrt{2\pi }e^{-\frac{1}{2}{\left(\varpi_{0}+s\varpi_{s}\right)}^{2}}e^{-i\varpi_{s}b}+\frac{b}{2}\sqrt{2\pi }e^{-\frac{1}{2}{\left(\varpi_{0}-s\varpi_{s}\right)}^{2}}e^{i\varpi_{s}b}$$

Then the instantaneous frequencies are computed using the next Equation [17]:

(22) $$\hat{f}(b)=\frac{c+\sqrt{c^{2}+2}}{2s_{\max}(b)}-2\pi f_{o}$$

where *f_o_* is the spatial frequency. At the end, the phase distribution can be extracted by integrating the estimated frequencies and no phase unwrapping algorithm is required.

## 3. Experimental Setup

**Figure 3.**
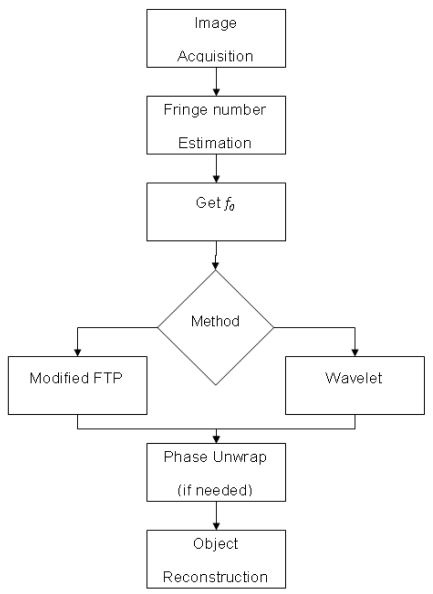
Proposed methodology.

The experimental setup shown in Figure 1 is used to apply the methodology proposed in Figure 3. The first step is to acquire the image. Due to the nature of the image, sometimes a filtering to eliminate the noise is necessary. In this research, only a 9 × 9 Gaussian filter is used in the Fourier transform. In the wavelet transform application, no filter is applied. Next, the number of fringes is estimated due to the fact that has a direct relationship with the fundamental frequency *fo*. Then, the *fo* is obtained by applying an algorithm. At this point, it is necessary to decide whether the Fourier or wavelet method would be applied. Both methods, Fourier and wavelet analysis use the algorithms described in the previous section.

When using the Fourier method, a robust unwrapping algorithm is needed, followed by an unwrapping algorithm with local and global analysis. The main algorithm for the local discontinuity analysis [5] is described as follows: (a) first, the wrapped phase map is divided into regions giving different weights (w_1_,w_2_, .., w_n_) to each region, (b) the modulation unit is defined that helps to detect the fringe quality and divides the fringes into regions, (c) regions are grouped from the biggest to the smallest modulation value, (d) next, the unwrapping process is started from the biggest to the smallest region, (e) later, an evaluation of the phase changes is carried out to avoid variations smaller than *fo*.

If the wavelet transform method is used then a simple unwrapping algorithm is enough to obtain the three-dimensional shape from the object. The final step is to obtain the object reconstruction and in some cases to determine the error (in case of virtual created objects).

In the experimental setup, a high-resolution digital CCD camera can be used. The reference plane can be any flat surface like a plain wall, or a whiteboard. In the reference plane it is important to consider a non reflective-surface to minimize the unwanted reflection effects that may cause some problems for the image acquisition process. The object of interest can be any three-dimensional object and for this work, three objects are considered (Figure 10); the first one is a mask, the second is an oval the third one is symmetrical pyramid.

It is also important to develop software able to produce several different fringe patterns. To create several patterns, it is necessary to modify the spatial frequency (number of fringes per unit area), and resolution (number of levels to create the sinusoidal pattern) of the fringe pattern. It may also be necessary to include into the software development a routine capable of performing phase shifting as well as to include the horizontal or vertical orientation projection of the fringe pattern.

### Computer Simulation

**Figure 4.**
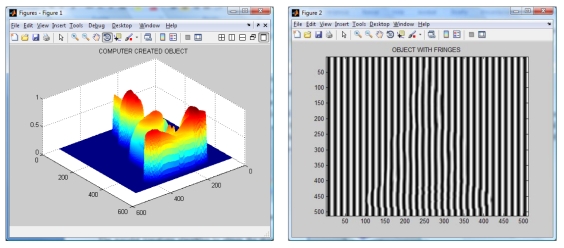
Computer created Buddha and fringes projected on it.

An object with a Buddha shape generated by computer is used to test the algorithms. The generated Buddha in shown in Figure 4. Then, a sinusoidal fringe pattern of known spatial frequency is created and later is added to the shape of the created object. The resulting image is shown in Figure 4. It is worth noting the distortions of the fringe pattern due to the object shape.

**Figure 5.**
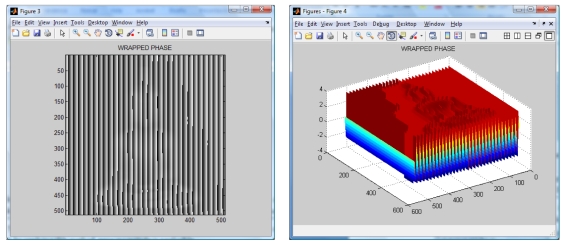
Wrapped phase (image and mesh).

**Figure 6.**
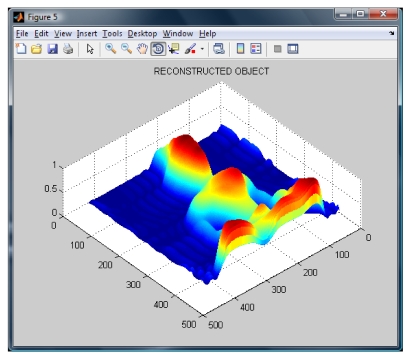
Reconstructed object using our Modified Fourier Transform Profilometry.

The wavelet transform algorithm is considered o obtain the shape of the Buddha. The resulting wrapped phase and its mesh are shown in Figure 5. The reconstructed Buddha using the modified Fourier Transform Profilometry can be seen in Figure 6. Notice that, by applying this method, the shape of the Buddha looks almost equal, however it has an error magnitude of about 3.5%.

**Figure 7.**
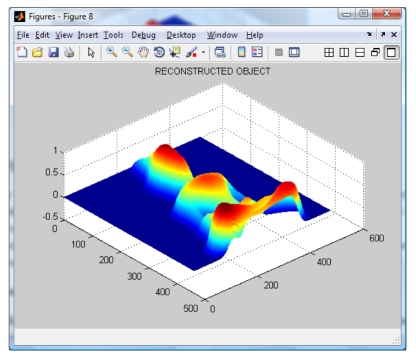
Reconstructed object using our Extended Wavelet Transform Profilometry.

By applying the Wavelet Profilometry, the mesh shown on Figure 7 is obtained; the whole volume presents an error of about 1.5%. At this point, it can be seen that even the amount of error seem to be almost equal for both methods, but the Fourier one is bigger and the final shape is more defined in the case of the wavelet method. The computer simulation allowed us to test and validate both methods.

As a preliminary conclusion, it is clear that the wavelet transform gives a better performance than the Fourier transform for the selected object shape. Therefore, the wavelet transform is selected and used. The error of the wavelet method is about 1 to 2% and using the Fourier one it is about 3 to 5%.

**Figure 10.**
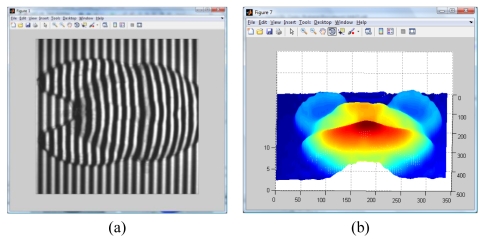

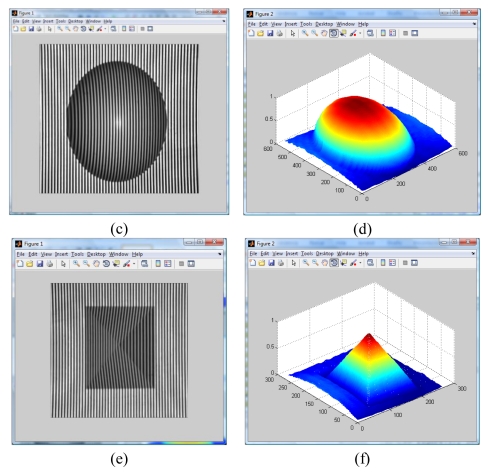
Real objects (a), (c), and (e), and their respective reconstruction (b), (d) and (f) views by using wavelet transform.

To validate the methodology, the following experiments were conducted. Several objects with different shapes were created by computer, where the height is known in every point in the object. Then, the Fourier and wavelet based analysis are applied as well as the methodology proposed by Gdeisat *et al.* [15]. The height of the virtual object was compared with each one of the analysis and the results are presented in Table 1. Finally, the wavelet method has been applied to several real objects, and the respective results of the reconstruction process can be seen in Figure 10.

## 4. Conclusions and Future Work

In this paper, a three-dimensional reconstruction methodology was presented. The method is based on the modified Fourier Transform Profilometry or Wavelet Transform Profilometry. In the first part of the proposed method the high frequencies that mostly affect the performance on the phase unwrapping in the Fourier method are obtained analytically. An object generated by the computer was virtually created and a known spatial sinusoidal fringe pattern was projected on it. Both Fourier and wavelet analysis were conducted, showing a good performance. In the comparison, the wavelet method was the one that showed a minimal error. Later, a real object was selected and the wavelet analysis was carried out and an accurate reconstruction of the object was achieved. This methodology could be widely used to digitize diverse objects for reverse engineering, virtual reality, 3D navigation, and so on.

Notice that the method can reconstruct only the part of the object that can be seen by the camera, if a full 3D reconstruction (360 degrees) is needed, a rotating table is can be used and the methodology will be applied *n* times, where *n* is the rotation angle of the table. As a future work, several tests could be carried out using objects with different shapes. Another option is to change the wavelet type and characterize the system.

One big challenge is to obtain the 3D reconstruction in real time. As a part of the solution, an optical filter could be implemented to obtain the FFT directly, or else, the algorithm can be implemented into a FPGA to carry out a parallel processing and minimize the processing time.

## Figures and Tables

**Figure 8.**
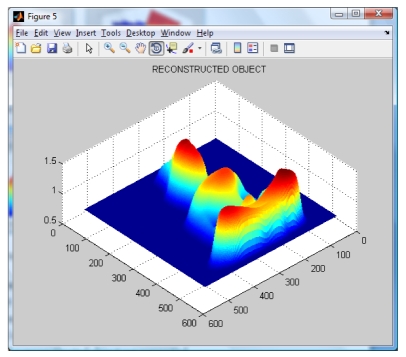
Reconstructed object using the methodology proposed by Gdeisat *et al.* [15].

**Figure 9.**
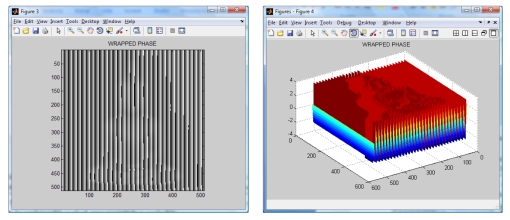
Wrapped phase (image and mesh).

**Table 1.** Error table.

**Object**	**Error (%)**		

	**Fourier**	**Wavelet**	**Gdeisat *et al.***
Buddha	3.499	1.455	1.901
Butterfly	3.776	1.733	1.824
Pyramid	4.871	1.923	2.054
